# Dysregulation of Neuronal Iron in Alzheimer’s Disease

**DOI:** 10.2174/1570159X20666211231163544

**Published:** 2023-09-01

**Authors:** Md. Sahab Uddin, Ghulam Md Ashraf

**Affiliations:** 1 Department of Pharmacy, Southeast University, Dhaka, Bangladesh;; 2 Pharmakon Neuroscience Research Network, Dhaka, Bangladesh;; 3 King Fahd Medical Research Center, King Abdulaziz University, Jeddah 21589, Saudi Arabia;; 4 Department of Medical Laboratory Technology, Faculty of Applied Medical Sciences, King Abdulaziz University, Jeddah 21589, Saudi Arabia

In the brain, iron (Fe) plays an important role in myelin synthesis, metabolism, and synthesis of neurotransmitters, and preserving the increased metabolic capacity of neurons [1, 2]. Iron metabolism also helps in maintaining brain homeostasis under normal physiological conditions [3]. If iron metabolism becomes impaired, it exerts deleterious actions on brain activities [4]. Pantothenate kinase-associated neurodegeneration is the most frequent form of neurodegeneration with brain iron accumulation (NBIA), a set of clinical ailments characterized by involuntary movements, muscle tone changes, and postural abnormalities [5]. Moreover, *PLA2G6* mutations (first found in atypical parkinsonism patients) cause infantile neuroaxonal dystrophy and are connected to NBIA [6, 7]. Sporadic neurodegenerative disorders like Alzheimer's disease (AD) have also been linked to high iron levels and iron-related pathogenic triggers [8]. In the brains of AD individuals, an elevated iron level in senile plaques (SPs) was observed [9]. Studies using a quantitative susceptibility map demonstrated the co-localization of amyloid-beta (Aβ) plaques and brain iron and also revealed the deposition of brain iron-mediated AD development [10]. In the brain, progressive iron deposition has also been found during the normal aging process (*i.e.* especially in the cortex, substantia nigra, caudate nucleus, and globus pallidus). These areas of the brain are closely linked with neurodegenerative disorders [11-13]. Deposition of iron in AD brain is more serious as compared to the healthy individuals of the same age. Iron metabolism is disrupted in AD, notably at the cellular iron efflux. In peripheral blood mononuclear cells of AD patients and healthy controls, Guerreiro *et al.* [14] looked at the expression of key iron metabolism-related genes, specifically those implicated in iron efflux. When comparing AD patients to healthy subjects, the expression of aconitase 1, ceruloplasmin and amyloid-beta precursor protein (APP) genes was shown to be significantly lower in AD patients. When peripheral blood mononuclear cells from AD patients were compared to controls, there was a substantial downregulation in the expression of genes related to ferroportin-mediated cellular iron export [14].

Iron metabolic disorders can stimulate Aβ generation and buildup [15]. The translation of APP mRNA can be selectively blocked by iron chelators [16, 17]. On the other hand, an elevated level of iron can augment the translation of APP mRNA and Aβ production [16, 17]. In APP/PS1E9 double transgenic mouse models, prolonged consumption of increased levels of iron increased the SP levels in the brain [18]. A study by Peters *et al.* [19] looked at how brain iron levels affect Aβ plaque burden and microgliosis over time. For 12 months, they were fed 3,5,5-trimethylhexanoyl ferrocene (TMHF, a lipophilic iron compound) and iron inadequate diets to knock-in APP mice. Compared to a control diet, TMHF increased brain iron by 22%, while iron insufficiency decreased brain iron by 21%. TMHF enhanced plaque development and senile plaques morphology. Moreover, increased plaque-iron burden and microglial iron additions were connected to higher brain iron levels [19].

Even though numerous studies confirmed that Aβ plaques and iron co-localize, but still it is now clear in what form iron exists in plaques. In a study using transmission electron microscopy, Plascencia-Villa *et al.* [20] confirmed that iron exists in the SPs core as iron oxide magnetite nanoparticles. It has also been confirmed that metal biology is linked with Aβ aggregation and iron accumulation. In an *in situ* study, it has been confirmed by X-ray magnetic circular dichroism that there is the presence of magnetite (*i.e*. an iron-oxide mineral) in human SPs [21]. Magnetite is not a typical characteristic of the human brain and raised level of this mineral suggests that impaired iron redox chemistry influences AD [22]. Telling *et al.* [23] in a study, found proof that amyloid plaque morphology and iron biochemistry are related in APP/PS1 mouse model of AD. This study found that when iron was reduced to a pure ferrous (Fe^2+^) state being linked to dense protein deposits in the APP/PS1 mouse cortex compared to wild-type mice [23]. The binding of Fe^2+^ to the peptide decreased the helix shape and increased the sheet content, suggesting that Fe^2+^ promotes oligomerization by increasing the peptide-peptide interaction. Another study has found in the absence of ferritin, and Aβ_1-42_ plays a role in forming magnetite nanoparticles in AD [24, 25]. Other than mediating Aβ aggregation, increased levels of iron can affect amyloidogenic APP processing. Initially, it has been observed that iron exerts modulatory activity on the α-secretase cleavage action of APP [26]. However, later studies revealed that furin regulates the mechanism of converting α- and β-secretases from the inactive status to the active status and iron can control furin expression at the transcriptional level [27]. Extremely high levels of iron can lead to the suppression of furin expression, which facilitates β-secretase activation, thus can mediate Aβ generation via the amyloid pathway [28]. Presenilin enhancer-2 also has the ability to bind with iron via ferritin light chain and can enhance activities of γ-secretase, thus can eventually result in increased Aβ generation [29]. A study found that the inert ferric core of ferritin was converted into more reactive low-oxidation states as a result of the co-aggregation of Aβ and ferritin [30].

Iron has been found to mediate *in vitro* Aβ aggregation and can also elevate their cytotoxic effects [31, 32]. Nonetheless, there are some arguments regarding the impact of iron and Aβ. Ferrous (Fe^3+^) and Fe^2+^ ions were found to interact with Aβ and APP in order to mediate Aβ aggregation into fibrous forms [33]. In addition to this, Fe^2+^ also interacts with amino acids of Aβ, which might trigger alterations in the amyloid form in a different way as compared to zinc and copper [34]. Whereas, Fe^3+^ easily binds with Aβ and gets reduced to Fe^2+^ and elevates the reactive oxygen species generation, which triggers β-secretase to cleave the monomer form of Aβ_1-42_ into more toxic oligomers, which further induces neuronal death [35, 36]. Indeed, Aβ can stimulate oxidative damage, convert Fe^2+^ to Fe^3+^ ion with redox action, and disrupt mitochondrial activity. These effects can further exacerbate iron overload and AD conditions [37, 38]. Moreover, iron exposure can mediate APP accumulation in cultured SHSY5Y cells, in consort with elevated β-secretase action and Aβ_1-42_ in the medium [39]. It has been revealed that treatment of neurons with iron-mediated the non-amyloidogenic APP pathway, this treatment also brought changes in soluble APPα distribution and its retention in cell lysates instead of its secretion outside the cell, whereas iron did not alter the expression of β-secretase, nonetheless considerably suppressed its effect [40]. In a different study, it was observed that Aβ could markedly decrease the redox capacity of iron, which might suggest neuroprotective activity and metal chelation of Aβ in the case of AD [41]. In the human brain, Everett *et al.* [42] found metallic Cu^0^ associated with Fe^0^. Synchrotron x-ray spectromicroscopy was used to identify such nanoscale biometal deposits within amyloid plaque cores of AD patients.

Another main AD pathological characteristic is neurofibrillary tangles (NFTs). Deposition of iron has also been found in neurons with NFTs [18]. Along with Aβ, iron can also bind with tau and can induce phosphorylation of tau, and phosphorylated tau aggregation, while this process can be reversed via iron chelators [43]. Interestingly, Fe^3+^causes hyperphosphorylated tau to aggregate, whereas the Fe^2+^ reverses the aggregation [44]. Collectively, these findings indicate that iron might contribute to the buildup of hyperphosphorylated tau to generate NFTs. In brain neurons, tau can indirectly participate in the iron ion transmission during AD pathogenesis [45]. Furthermore, studies revealed that iron plays role in tau hyperphosphorylation via activating glycogen synthase kinase-3β (GSK-3β) and cyclin-dependent kinase 5 (CDK5)/P25 complex [46, 47], however, no related studies have confirmed whether iron also plays roles in the protein phosphatase 2A inactivation. Tau nitration averts its microtubule stabilization in case of AD, and nitrating tau was seen in SPs and tau entanglement [48, 49]. In NFTs, tau accumulation is also linked with elevated heme oxygenase-1 (HO-1, a potent antioxidant) induction [50]. Indeed, HO1 has the ability to metabolize heme secreted by damaged mitochondria, and HO1 can also mediate Fe^2+^ release, which might result in free radicals to start additional oxidative damage [28]. Therefore, iron-mediated oxidative damage might mediate tau hyperphosphorylation and its aggregation. Cross-sectional and longitudinal studies suggested that AD patients had higher iron concentrations in the deep gray matter and neocortical areas than healthy control subjects [51].

Iron as a target in the development of AD has been a research focus in recent years. Iron chelators can be used to chelate excess iron in the brains of AD patients. Several putative agents targeting different factors of iron dysregulation are now being explored in various clinical and preclinical studies. In the future, newly identified mechanisms such as ferroptosis might be interesting targets for AD treatment.

## LIST OF ABBREVIATIONS

AD: Alzheimer's Disease
APP: Amyloid-beta Precursor Protein
Aβ: Amyloid-beta
NFTs: Neurofibrillary Tangles
SPs: Senile Plaques
